# Application of Convolutional Neural Network-Based Feature Extraction and Data Fusion for Geographical Origin Identification of Radix Astragali by Visible/Short-Wave Near-Infrared and Near Infrared Hyperspectral Imaging

**DOI:** 10.3390/s20174940

**Published:** 2020-09-01

**Authors:** Qinlin Xiao, Xiulin Bai, Pan Gao, Yong He

**Affiliations:** 1 College of Biosystems Engineering and Food Science, Zhejiang University, Hangzhou 310058, China; qinlxiao@zju.edu.cn (Q.X.); xlbai@zju.edu.cn (X.B.); 2 Key Laboratory of Spectroscopy Sensing, Ministry of Agriculture and Rural Affairs, Hangzhou 310058, China; 3 College of Information Science and Technology, Shihezi University, Shihezi 832000, China; gp_inf@shzu.edu.cn

**Keywords:** Radix Astragali, hyperspectral imaging, geographical origin, convolutional neural network, data fusion

## Abstract

Radix Astragali is a prized traditional Chinese functional food that is used for both medicine and food purposes, with various benefits such as immunomodulation, anti-tumor, and anti-oxidation. The geographical origin of Radix Astragali has a significant impact on its quality attributes. Determining the geographical origins of Radix Astragali is essential for quality evaluation. Hyperspectral imaging covering the visible/short-wave near-infrared range (Vis-NIR, 380–1030 nm) and near-infrared range (NIR, 874–1734 nm) were applied to identify Radix Astragali from five different geographical origins. Principal component analysis (PCA) was utilized to form score images to achieve preliminary qualitative identification. PCA and convolutional neural network (CNN) were used for feature extraction. Measurement-level fusion and feature-level fusion were performed on the original spectra at different spectral ranges and the corresponding features. Support vector machine (SVM), logistic regression (LR), and CNN models based on full wavelengths, extracted features, and fusion datasets were established with excellent results; all the models obtained an accuracy of over 98% for different datasets. The results illustrate that hyperspectral imaging combined with CNN and fusion strategy could be an effective method for origin identification of Radix Astragali.

## 1. Introduction

Radix Astragali, the dried root of *Astragalus*, is a prized traditional Chinese functional food that is used for both medicine and food purposes, especially in Korea, China, and Japan [1,2]. The nutritional composition of Radix Astragali includes polysaccharide, flavonoids, saponin, alkaloids, amino acids, and various mineral elements [3]. Radix Astragali has been proven to provide multiple health functions such as immunomodulation, anti-tumor, and anti-oxidation [1,4,5,6]. With the development of society and the improvement in people’s living standard, the global demand for Radix Astragali has gradually increased with a corresponding increase in the planted areas of Radix Astragali. The functional components are closely related to its health functions. The content of the functional components of Radix Astragali varies due to differences in environmental conditions such as climate and soils. Therefore, the identification of Radix Astragali from different geographical origins is essential for quality evaluation.

Although the geographical origins of Radix Astragali can be discerned by experienced farmers and experts, this method is time-consuming and laborious. Chemical analysis methods such as ultra-high-performance liquid chromatography (UHPLC) are powerful tools for identifying the origin of Radix Astragali [7,8]. However, these methods are time-consuming and destructive, and require complex pre-processing and highly skilled operators. In addition, these approaches are only applicable under laboratory conditions, which means they are inefficient and are difficult to conduct on a large-scale.

Computer vision, as a non-chemical and non-destructive technique, has advantages in identifying samples with significant differences in their external characteristics. Although computer vision offers good recognition of changes in morphology and texture, it is unable to obtain information about the internal composition. The unique advantage of near-infrared spectroscopy is that it can obtain spectral information associated with the internal composition of samples and has been widely used to identify the attributes of agricultural products including the variety and geographical origin [9,10,11]. However, the near-infrared spectroscopy cannot obtain spatial information for the entire sample. Hyperspectral imaging (HSI), allows the acquisition of one-dimensional spectral information and two-dimensional image information, and provides a comprehensive analysis of samples. In the past few years, HSI has gained increasing attention for quality assessment and variety classification in the field of agriculture [12,13,14], food [15], and traditional Chinese functional food [16,17]. Among studies that have utilized hyperspectral imaging to classify traditional Chinese functional food, Ru et al. [18] proposed a data fusion approach in the Vis-NIR and short-wave infrared spectral range, and obtained a classification accuracy of 97.3% for the geographical origins of *Rhizoma Atractylodis Macrocephalae*. Xia et al. [19] investigated the effect of wavelength selection methods on the identification of *Ophiopogon Japonicus* of different sources, and the optimal accuracy was 99.1%. These studies demonstrate the feasibility of using hyperspectral imaging to identify the geographical origins of samples.

Effective analysis of the massive amount of data acquired by HSI is a great challenge that hinders its application. At present, machine learning methods and deep learning approaches have been developed and are regarded as ideal options for processing and analyzing hyperspectral images. Convolutional neural network (CNN) is a novel deep learning approach, which has the abilities of self-learning, feature extraction, and large-scale data handling. CNN achieves rapid and efficient data analysis by building networks composed of a large number of neurons. Given that CNN has the unique power of self-learning and excellent performance, CNN has been widely welcomed by researchers and has been applied for processing spectra and hyperspectral images [20,21], as well as remotely-sensed data [22].

Fusion strategy has gained attention in realizing comprehensive analyses, because it is able to integrate information obtained from different sources. The superiority of fusion strategy in food quality authentication has been proved [23]. However, in the application of hyperspectral imaging, there are few studies that have reported the use of fusion strategy for spectra and corresponding features from hyperspectral images at different spectral ranges. As far as we know, no studies have reported the application of HSI in the identification of the geographical origin of Radix Astragali. Therefore, the aim of this study was to investigate the feasibility of using HSI at two different spectral ranges, CNN and data fusion to discriminate the geographical origin of Radix Astragali. The specific objectives include (1) explore the feasibility of identifying Radix Astragali from five geographical origins using hyperspectral imaging at a visible/short-wave near-infrared range (Vis-NIR, 380–1030 nm) and near-infrared range (NIR, 874–1734 nm); (2) extract the PCA score features and deep spectral features to represent the original spectra by PCA and CNN, respectively; (3) build SVM, LR, and CNN classification models based on full wavelengths, PCA score features, and deep spectral features to quantitatively identify Radix Astragali from five geographical origins; (4) develop prediction maps of Radix Astragali from five geographical origins in the Vis-NIR and NIR spectral range; and (5) build SVM, LR, and CNN classification models based on measurement-level and feature-level fusion datasets of Radix Astragali from five geographical origins.

## 2. Materials and Methods

### 2.1. Sample Preparation

**Figure 1:**
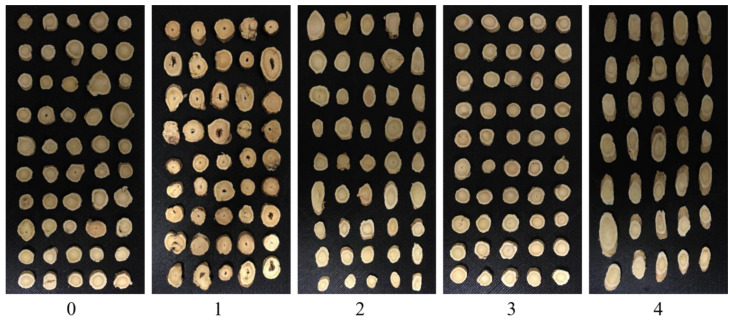
RGB images of Radix Astragali from Gansu province, Heilongjiang province, the Inner Mongolia autonomous region, Shanxi province, and the Xinjiang Uygur autonomous region.

Radix Astragali from five different geographical origins were collected in 2019, including Gansu province, Heilongjiang province, the Inner Mongolia autonomous region, Shanxi province, and the Xinjiang Uygur autonomous region, China. The collected Radix Astragali has already been sliced, and the thickness of all samples was about 3 mm (as shown in Figure 1). Due to the different slicing methods in the initial processing, the shape of the Radix Astragali from different geographical origins is slightly different. The number of samples from Gansu province, Heilongjiang province, the Inner Mongolia autonomous region, Shanxi province and the Xinjiang Uygur autonomous region was 1040, 568, 840, 1032 and 648, respectively. For further analysis, the category of the Radix Astragali was assigned as 0, 1, 2, 3, and 4 (corresponding to the geographical origins of Gansu province, Heilongjiang province, the Inner Mongolia autonomous region, Shanxi and the Xinjiang Uygur autonomous region, respectively).

### 2.2. Hyperspectral Image Acquisition

Hyperspectral images were acquired by two hyperspectral imaging systems, which covered the spectral range of 380–1030 nm and 874–1734 nm, respectively. Detailed information about the two hyperspectral imaging systems can be found in the literature [24].

Radix Astragali was placed on a black plate and moved into the imaging field on the platform. To avoid distortion and a deformed hyperspectral image, the moving speed of the mobile platform, the distance between the camera lens and the samples, and the camera exposure time are required to be appropriately set. For the visible/short-wave near-infrared hyperspectral imaging system, these three parameters were adjusted to 0.95 mm/s, 16.8 cm, and 0.14 s, respectively. For the near-infrared hyperspectral imaging system, the same three parameters were set to 12 mm/s, 15 cm, and 3 ms, respectively.

The correction procedure should be conducted on the raw images. The reflectance image was corrected using the following equation:

(1) $$I_{C}=\frac{I_{raw}-I_{dark}}{I_{white}-I_{dark}}$$

where *I_c_*, *I_raw_*, *I_white_* and *I_dark_* are the calibrated hyperspectral image, raw hyperspectral image, the white and dark reference image, respectively. The white reference image was acquired by using a white Teflon board with a reflectance of about 100%. The dark reference image was obtained by covering the camera lens completely with a reflectance of about 0%.

### 2.3. Image Preprocessing and Spectral Extraction

The segmentation between Radix Astragali and the background is crucial for accurately extracting spectral information. Firstly, every single piece of Radix Astragali was defined as the region of interest (ROI). A mask was built by conducting image binarization on the gray-scale image at 989 nm and 1119 nm for Vis-NIR images and NIR images, respectively. The mask was used to remove background information. Pixel-wise spectra within each ROI were extracted. The head-to-tail spectra with high random noise levels were first removed. The spectral range of 441–947 nm (400 bands) for Vis-NIR HSI and 975–1646 nm (200 bands) for NIR HSI were used for analysis. For Vis-NIR spectra, the pixel-wise spectra were preprocessed using wavelet transform of Daubechies 8 with a decomposition scale of 3. For NIR spectra, the pixel-wise spectra were preprocessed using wavelet transform of Daubechies 9 with a decomposition scale of 3. Then, the pixel-wise spectra of all pixels within each Radix Astragali sample were averaged as one spectrum.

### 2.4. Data Analysis Methods

#### 2.4.1. Principal Component Analysis

Principal component analysis (PCA) is a powerful statistical method, which is commonly used for data dimensionality reduction and feature extraction. PCA obtains the main variables with a high interpretation rate by projecting the original variables into new orthogonal variables. These new variables are called principal components (PCs). Generally, the first few PCs with a high interpretation rate contain most of the original variable information, which contributes to the full representation of the original high-dimensional matrix. PCA can be used for the qualitative identification and clustering of samples.

For hyperspectral images, PCA of pixel-wise and object-wise was conducted [25]. Score images under different PCs can be formed by pixel-wise analysis. Hyperspectral images of Radix Astragali from five geographical origins were randomly selected to calculate PCA. By bringing the pixel-wise spectra that had been preprocessed by wavelet transform into the PCA, the pixel-wise score values under different PCs can be obtained. The differences or similarities between samples are reflected in the score images of these PCs.

Object-wise analysis was applied on the average spectra of all samples, instead of individual pixels, for feature extraction. The scores containing the most information about the spectral characteristics were used as input features for the classifiers. In our study, the first 20 principal component scores were used as extracted features.

#### 2.4.2. Convolutional Neural Network

Convolutional neural network (CNN) is a feedforward neural networks that involves convolution calculations. CNN has powerful learning capabilities, and this model is also widely used as an algorithm of deep learning [24,26].

**Figure 2:**
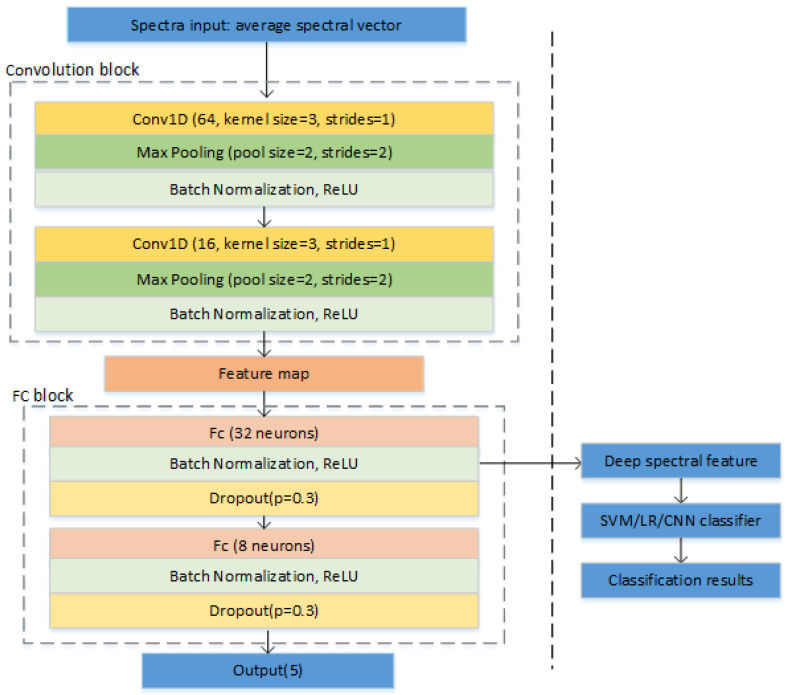
The structure of the convolutional neural network (CNN) and the flowchart of deep spectral features used in the support vector machine (SVM)/logistic regression (LR)/CNN classifier.

A simple CNN architecture was designed for our study, which was applied for deep spectral feature extraction. The structure of the CNN and the flowchart of deep spectral features used in the classifiers are shown in Figure 2. The first block was a convolution block, which included two convolutional layers. Both of the convolutional layers were followed by a max pooling layer and a batch normalization layer. Since the analysis object was one-dimensional spectral data, one-dimensional convolution was applied [27]. The parameters of both convolutional layers were set at a kernel size of 3 and strides of 1 without padding. The number of filters was 64 and 16 for the first and second convolutional layers, respectively. The rectified linear unit (ReLU) served as an activation function. The second block was a fully connected (FC) network. Two FC layers were included, and they consisted of 32 and 8 neurons, respectively. A batch normalization layer and a dropout layer followed the FC layer in turn. The dropout layer was utilized to alleviate over-fitting, and the dropout ratio was set as 0.3. At the end of the network, another dense layer was added for output.

A Softmax cross-entropy loss function integrated with a stochastic gradient descent (SGD) optimizer was employed to train the proposed CNN. A scheduled learning rate was used, and the batch size was fixed as 128. At the beginning of the training, a relatively large learning rate of 0.5 was used to speed up the training process. The learning rate was reduced to 0.1 and 0.05 when the accuracy on the validation set exceeded 0.8 and 0.9, respectively. The training process was terminated once the accuracy of the validation set reached 0.95. For both spectral ranges, the output of the first batch normalization layer in the FC block was extracted as deep spectral features. Thus, 32 deep spectral features were used as input in classifiers for realizing the classification of Radix Astragali from different geographical origins.

In addition, because of CNN’s powerful classification abilities [28], it was also used to build classification models.

#### 2.4.3. Data Fusion Strategy

To achieve a more comprehensive and reliable analysis, fusion strategies were developed [23]. In this study, two hyperspectral imaging systems covering different spectral ranges were used. The spectra acquired by different HSI systems cannot be connected directly. To fully mine useful information, two different fusion strategies (measurement-level fusion and feature-level fusion) were adopted to investigate the feasibility of using different fusion datasets for the classification of Radix Astragali from different geographical origins.

For the measurement-level fusion, the original spectra obtained by two sensors (visible/short-wave near-infrared hyperspectral imaging system and near-infrared hyperspectral imaging system) were concatenated as a new matrix. For the feature-level fusion, feature extractors were utilized to process the original spectra obtained from different sensors. Then the derived features were combined as the input for subsequent analysis. PCA and CNN were used as feature extractors.

#### 2.4.4. Traditional Discriminant Model

Support vector machine (SVM), a pattern recognition algorithm, is widely used in linear classification [29]. SVM classifies different samples by exploring the hyperplane that maximizes the distance between different categories. The kernel function is crucial for the training of SVM. The radial basis function (RBF) has been widely used in spectral analysis due to its ability to handle nonlinear problems [30]. In this study, the RBF kernel was used. The optimal values of regularization parameter *c* and kernel function parameter *g* were optimized by grid-search. The searching range of *c* and *g* were assigned as 2^−8^ to 2^8^.

Logistic regression (LR) is a generalized linear regression analysis model, which is commonly used to deal with classification problems. The sigmoid function is added based on linear regression, which is used to make the output value of LR in the range of 0 to 1. The output value refers to the probability of determining the sample as a specific class [14]. LR realizes the output of the classification probability value through nonlinear mapping. The parameters of *optimize_algo*, the regularization term *r*, and the regularization coefficient *c*′ are essential for LR models. In this study, the optimal combination of the parameters was adjusted by grid-search, and a ten-fold cross-validation was carried out. The searching range of *optimize_algo*, *r* and *c*′ were assigned as (newton-cg, lbfgs, liblinear and sag), (L1, L2) and (2^−8^–2^8^).

#### 2.4.5. Model Evaluation

It is of great importance to evaluate the performance of discriminant models by using appropriate indicators. Classification accuracy was calculated as the ratio of the number of the correctly classified samples to the total number of samples.

To better evaluate the performance and stability of the models, the original dataset of samples was randomly divided five times into a calibration set, validation set, and prediction set at a ratio of 6:1:1. Thus, five calibration sets, validation sets, and prediction sets were obtained. All the models were built for these datasets. The average accuracy and standard deviation of the five calibration sets were calculated, and the same calculation was also conducted for the validation set and prediction set. For a robust model, the average accuracy should be close to 100%, and the standard deviation should be as small as possible.

#### 2.4.6. Significance Test

Least significant difference tests were applied to the compare the accuracy obtained by the SVM, LR and CNN models at a significance level of 0.05.

#### 2.4.7. Software

The hyperspectral images were firstly preprocessed in ENVI 4.7 (ITT Visual Information Solutions, Boulder, CO, USA) to define the area of the samples. Matlab R2019b (The Math Works, Natick, MA, USA) is a powerful mathematical calculation software. Pixel-wise spectra extraction, pixel-wise PCA and object-wise PCA were conducted in Matlab R2019b. SVM, LR, and the corresponding grid-searches were undertaken in the scikit-learn 0.23.1 (Anaconda, Austin, TX, USA) using python 3.1. CNN architecture was built in MXNet1.4.0 (MXNetAmazon, Seattle, WA, USA). Least significant difference tests were conducted in SPSS V19.0 software (The SPSS Inc., Chicago, IL, USA).

## 3. Results and Discussion

### 3.1. Overview of Spectral Profiles

**Figure 3:**
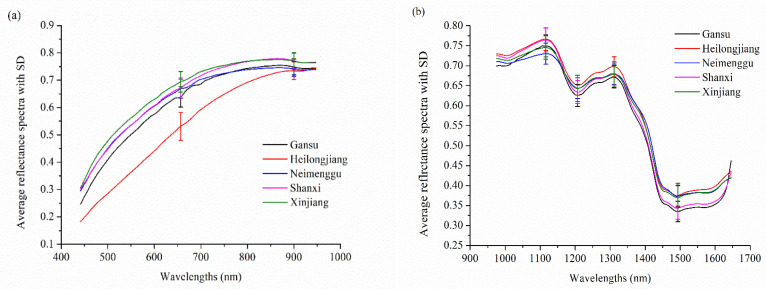
The average spectra with standard deviation (SD) of Radix Astragali from five origins in the range of (**a**) 441–947 nm (the standard deviation at 657 and 900 nm are shown); (**b**) 975–1646 nm (the standard deviation at 1116, 1207, 1311 and 1494 nm are shown).

**Figure 4:**
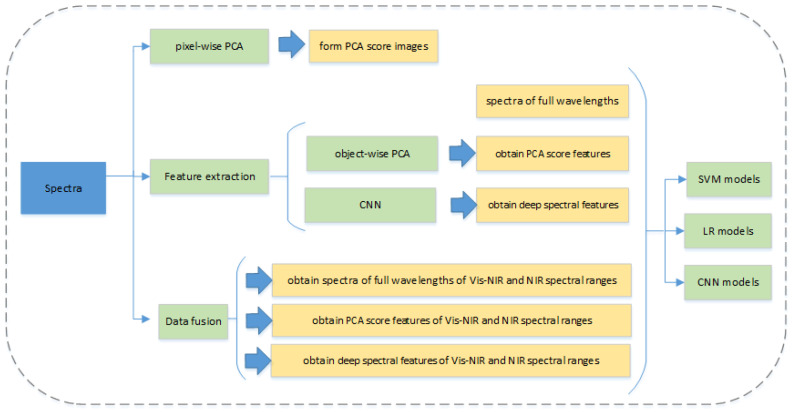
The flow chart of the analysis process in this study.

Figure 3a,b show the average spectra with the standard deviation of Radix Astragali from five different geographical origins at the Vis-NIR spectral range (441–947 nm) and NIR spectral range (975–1646 nm), respectively. As shown in Figure 3a, the spectral curves in 441–947 nm present a consistent upward trend with the increase in wavelengths and level off at 900 nm. The spectral difference of Radix Astragali from different geographical origins at certain wavelengths was observed. The spectra curves in the NIR spectral range are displayed in Figure 3b, and similar trends were observed in the spectral curves of Radix Astragali from different geographical origins. Two peaks (1116 nm and 1311 nm) and two valleys (1207 nm and 1494 nm) were found in all of the spectral curves. The peaks and valleys are associated with the overtones and the combination of the bond vibrations (e.g., C-H, N-H, and O-H groups) in the constituent molecules [31]. The peak at around 1116 nm is designated approximately as the second overtone of C-H stretching vibrations of carbohydrates [32]. The spectral valley near 1207 nm is attributed to the plane bend of O-H [33]. The peak around 1311 nm is attributed to the first overtone of O-H stretch, and the valley near 1494 nm is designated as the first overtone of the asymmetric and symmetric N-H stretch [34]. Although there are some differences in the reflectance in specific spectra range, overlaps exist between these spectral curves. Therefore, it is necessary to conduct further analysis to identify Radix Astragali of different geographical origins. The general data analysis procedure of this study is presented in Figure 4.

### 3.2. PCA Score Images

**Figure 5:**
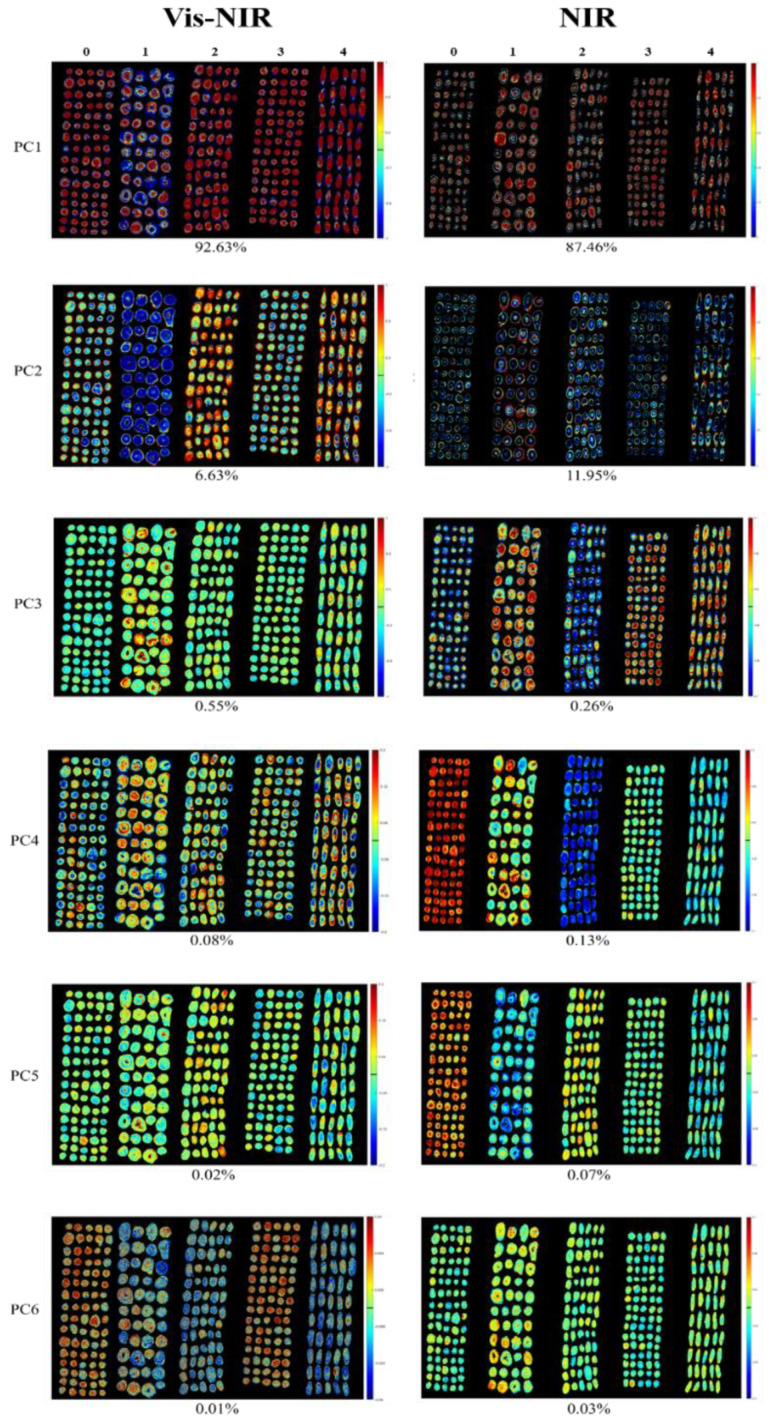
Principal component analysis (PCA) score images of the first six PCs for Radix Astragali from five geographical origins. (Left side: PCA score images of hyperspectral images in the Vis-NIR region; Right side: PCA score images of hyperspectral images in the NIR region).

Vis-NIR hyperspectral images and NIR hyperspectral images of Radix Astragali from five geographical origins were randomly selected for pixel-wise PCA. Score images were formed for each PC. Radix Astragali from different origins could be qualitatively discriminated through the difference in the colors. More than 99.9% of the information in the original spectra was concentrated in the first six PCs, which reflected 99.92% and 99.91% of the information in the original spectra for the Vis-NIR hyperspectral image and NIR hyperspectral image, respectively. The PCA score images of Radix Astragali from five geographical origins are shown in Figure 5. Zero, 1, 2, 3, and 4 represent the samples from Gansu province, Heilongjiang province, the Inner Mongolia autonomous region, Shanxi and the Xinjiang Uygur autonomous region, respectively. The color bar corresponds to the score values of the pixels. For samples with higher scores, more red pixels can be observed, while those with lower values correspond to more green and blue pixels.

As shown in the left column of Figure 5, for the Vis-NIR hyperspectral image, the first two PCs explained more than 99% of the original spectral information, while the other four PCs showed a less than 1% interpretation rate. Although these PCs showed less data viability, they still contained some information related to the samples. As illustrated above, Radix Astragali from different geographical origins could be qualitatively distinguished by different colors. In the PC1 image, Radix Astragali from Heilongjiang province had slightly lower score values than that of samples from the other four origins and showed the bluest color. In the PC2 image, the color differentiation between samples was more obvious. Similar to the results in the PC1 image, Radix Astragali from Heilongjiang province had the lowest score. Radix Astragali from the Inner Mongolia autonomous region had the highest score, and the color was partially red. The score values of samples from the Xinjiang Uygur autonomous region were slightly lower than those from the Inner Mongolia autonomous region, and the samples were less red than samples from the Inner Mongolia autonomous region. In addition, Radix Astragali from Gansu province showed relatively more blue pixels than that from Shanxi province. Furthermore, PC5 and PC6 images also proved the feasibility of making a rough distinction between samples from different origins.

The NIR hyperspectral images are shown in the right column of Figure 5. PC1 and PC2 carried most of the variance information, with 87.46% for PC1 and 11.95% for PC2. However, the samples of different origins cannot be intuitively distinguished in PC1 and PC2 images. This phenomenon may be because the information in the PC1 and PC2 images only represent the general characteristics of Radix Astragali. On the contrary, Radix Astragali from different origins show apparent color differences in PC4 images, although PC4 explained only 0.13% of the original spectral information. It can be seen that Radix Astragali from Gansu province had the highest score and presented the reddest color, followed by Heilongjiang province, Shanxi province, and the Xinjiang Uygur autonomous region, with the color gradually turned blue. Samples from the Inner Mongolia autonomous region could be further identified because they had the lowest score and a blueish color. The results indicated that besides the first three PCs, even though the rest of the PCs explained little of the data viability, they contained useful information that is conducive to identifying the geographical origins of Radix Astragali.

### 3.3. Discrimination Results of Models Using Full Wavelengths and Extracted Features

The SVM, LR, and CNN models were built based on full wavelengths, PCA score features, and deep spectral features. The results are shown in Table 1, Table 2 and Table 3.

Table 1 shows the classification results of the SVM, LR, and CNN models based on full wavelengths in the Vis-NIR spectral range and NIR spectral range. All the models based on full wavelengths in the Vis-NIR spectral range and NIR spectral range obtained decent results, with the average accuracy exceeding 98%. Overall, the models built based on Vis-NIR spectra were similar to those based on NIR spectra. Also, regardless of the spectral range, the results obtained by the CNN models were more satisfactory than the SVM and LR models, with an average accuracy close to 100% for the calibration, validation and the prediction set. Moreover, the average accuracy of the calibration set, validation set, and prediction set for CNN models was mostly significantly different to that of the SVM and LR models. Besides, compared to the SVM and LR models, the standard deviation for the CNN models was relatively lower in most cases, illustrating the stability of the CNN model.

Table 2 demonstrates the classification results of the SVM, LR, and CNN models based on the PCA score features of the Vis-NIR spectral range and NIR spectral range. Most of the average accuracy for the models built in the NIR spectral range was slightly higher than that in Vis-NIR spectral range. Although the average accuracy of the SVM, LR, and CNN models was very close, the significance test showed that there were still some differences. The performance of the LR models on the validation set was better, with an average accuracy of 99.380% and 99.806% for the Vis-NIR spectral range and NIR spectral range, respectively, which was significantly different to the SVM and CNN models in both spectral ranges.

The results of the classification models based on deep spectral features of the Vis-NIR spectral range and NIR spectral range are shown in Table 3. It can be observed that all the models based on deep spectral features performed significantly well, and all the average accuracies were over 99% in both spectral ranges. For the Vis-NIR spectral range, the average accuracy for the prediction set of all models was between 99.496–99.535%, which was slightly inferior to that for the NIR spectral range, which was within the range of 99.767–99.884%. The average accuracy of the CNN model for the prediction set was similar to other models, demonstrating the feasibility of combining deep spectral features and CNN for Radix Astragali classification.

The performance of the models based on PCA score features and deep spectral features was quite similar to that found in models based on full wavelengths. As shown in Table 1, Table 2 and Table 3, the average accuracy of the prediction set was in the range of 98.682–99.453% and 99.496–99.884% for models built on PCA score features and deep spectral features, respectively, which were quite close, even slightly better than that of models built on full wavelengths. This phenomenon exhibited the enormous potential of using characteristics to realize the accurate classification and reduce the heavy computational complexity.

### 3.4. Prediction Maps

The above results revealed that the geographical origin of Radix Astragali could be identified quickly and effectively by HSI. The performance of the CNN model based on full wavelengths in the prediction set was slightly superior to the other models in both spectral ranges. Therefore, the CNN model based on full wavelengths was utilized for form prediction maps for Radix Astragali from different geographical origins. By using spectral data, the prediction value of each pixel could be obtained, corresponding to the category value. Thus, the prediction map was formed.

**Figure 6:**
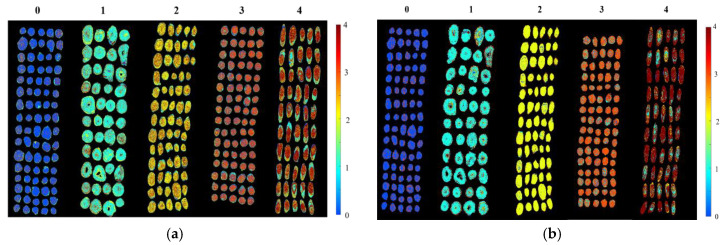
Prediction map for Radix Astragali of all the geographical origins based on the CNN model using full wavelengths: (**a**) prediction map using Vis-NIR spectra; and (**b**) prediction map using NIR spectra.

Figure 6 shows the prediction maps for Radix Astragali from five geographical origins obtained by spectra at two different ranges. The color bar represents the category values by colors. In this study, 0, 1, 2, 3, and 4 represent samples from Gansu province, Heilongjiang province, the Inner Mongolia autonomous region, Shanxi province, and the Xinjiang Uygur autonomous region, respectively. As shown in the prediction maps, the category value of each Radix Astragali sample was visualized, and the Radix Astragali sample with the same colors belonged to the same class (corresponding to the same geographical origin). Almost all the samples were correctly predicted with the corresponding category values. Thus, Radix Astragali from different geographical sources could be clearly distinguished in the prediction maps.

### 3.5. Discrimination Results of Models Using Fusion Strategy

The effect of three different fusion approaches (measurement-level fusion, PCA score feature-level fusion, and deep spectral feature-level fusion) was evaluated. The results of the classification models based on the dataset obtained by fusion strategies are shown in Table 4.

For the measurement-level fusion, the dataset integrated spectra variables in both the Vis-NIR and NIR ranges. The average accuracy for the calibration set, validation set, and prediction set was over 99%. The standard deviation ranged from 0.018–0.402%. Compared to the performance of the models based on spectra obtained by a single sensor, the fusion method improved the classification results and their stability. For the feature-level fusion, PCA score features from both the Vis-NIR and NIR ranges were combined, as well as deep spectral features. The accuracy for the classification sets of all models was over 99%. The excellent performance of the models demonstrated the potential of deep spectral features to represent the original data information, which has also been confirmed by previous studies [14,35,36,37,38]. Although the excellent classification of the original data has already been achieved, feature extraction and fusion strategy also obtained similarly outstanding results, which indicates that these are feasible strategies that could be explored further in future research.

On the whole, the overall accuracy was superior to the accuracy of 96.43% of previous study [39], which used a fusion of Raman spectra and ultraviolet-visible absorption spectra to classify different geographical origins of Radix Astragali.

## 4. Conclusions

This study investigated the feasibility of applying HSI coupled with CNN and a data fusion strategy to determine the geographical origin of Radix Astragali. Hyperspectral images in Vis-NIR range (380–1030 nm) and NIR range (874–1734 nm) were acquired. Score images formed as a result of pixel-wise PCA provided a good illustration of the diversity of the different geographical sources of Radix Astragali. Excellent results were obtained for all of the classification models based on full wavelengths and extracted features, with an average accuracy of over 98% for all classification sets. The features extracted from PCA and CNN were highly representative of the original spectra, and the models based on features extracted from PCA and CNN obtained similar, even better results than those based on full wavelengths. The overall results demonstrated that HSI coupled with CNN and a data fusion strategy is a powerful approach for determining the geographical origin of Radix Astragali, and allows the rapid and non-destructive identification of samples from different geographical origins.

## Figures and Tables

**Table 1:** The results of the classification models based on full wavelengths.

Spectra	Models	Cal	Val	Pre
Vis-NIR	SVM	99.477 ± 0.243 ^a^	98.295 ± 0.572 ^b^	98.953 ± 0.446 ^b^
	LR	98.418 ± 0.171 ^b^	98.101 ± 0.704 ^b^	98.798 ± 0.677 ^b^
	CNN	99.606 ± 0.368 ^a^	99.961 ± 0.087 ^a^	99.961 ± 0.087 ^a^
NIR	SVM	99.785 ± 0.105 ^b^	99.109 ± 0.524 ^b^	98.837 ± 0.930 ^b^
	LR	99.535 ± 0.067 ^c^	99.264 ± 0.442 ^ab^	99.263 ± 0.913 ^ab^
	CNN	99.994 ± 0.014 ^a^	99.806 ± 0.237 ^a^	99.923 ± 0.105 ^a^

The letters (a, b, c) in each column indicate the significance in the difference in the accuracy of different models at the confidence level of 5%. Within a column, data followed by different letters are significantly different.

**Table 2:** The results of the classification models based on PCA score features.

Spectra	Models	Cal	Val	Pre
Vis-NIR	SVM	99.238 ± 0.286 ^b^	98.295 ± 0.634 ^b^	98.835 ± 0.566 ^a^
	LR	99.548 ± 0.082 ^b^	99.380 ± 0.442 ^a^	99.380 ± 0.347 ^a^
	CNN	99.780 ± 0.053 ^a^	98.527 ± 0.652 ^b^	98.682 ± 0.860 ^a^
NIR	SVM	99.703 ± 0.090 ^b^	99.264 ± 0.420 ^b^	98.760 ± 0.884 ^a^
	LR	99.968 ± 0.000 ^a^	99.806 ± 0.237 ^a^	99.453 ± 0.912 ^a^
	CNN	99.890 ± 0.054 ^a^	98.953 ± 0.221 ^b^	98.760 ± 0.446 ^a^

The letters (a, b) in each column indicate the significance in the difference in the accuracy of different models at the confidence level of 5%. Within a column, data followed by different letters are significantly different.

**Table 3:** The results of the classification models based on deep spectral features.

Spectra	Models	Cal	Val	Pre
Vis-NIR	SVM	99.612 ± 0.208 ^a^	99.186 ± 0.373 ^a^	99.496 ± 0.294 ^a^
	LR	99.683 ± 0.136 ^a^	99.109 ± 0.591 ^a^	99.496 ± 0.402 ^a^
	CNN	99.729 ± 0.132 ^a^	99.070 ± 0.373 ^a^	99.535 ± 0.221 ^a^
NIR	SVM	100.000 ± 0.000 ^a^	99.845 ± 0.087 ^a^	99.884 ± 0.106 ^a^
	LR	99.994 ± 0.014 ^a^	99.806 ± 0.137 ^a^	99.767 ± 0.213 ^a^
	CNN	99.974 ± 0.027 ^a^	99.767 ± 0.212 ^a^	99.806 ± 0.237 ^a^

The letters in each column indicate the significance in the difference in the accuracy of different models at the confidence level of 5%. Data followed by the same letter are not significantly different.

**Table 4:** The results of the classification models based on fusion strategies.

Fusion Strategy	Models	Cal	Val	Pre
measurement fusion	SVM	99.903 ± 0.069 ^b^	99.147 ± 0.378 ^b^	99.535 ± 0.324 ^a^
	LR	99.852 ± 0.029 ^b^	99.612 ± 0.137 ^a^	99.690 ± 0.402 ^a^
	CNN	99.981 ± 0.018 ^a^	99.845 ± 0.087 ^a^	99.922 ± 0.174 ^a^
PCA feature fusion	SVM	99.897 ± 0.058 ^b^	99.186 ± 0.373 ^b^	99.535 ± 0.324 ^a^
	LR	99.987 ± 0.029 ^a^	99.961 ± 0.087 ^a^	99.767 ± 0.318 ^a^
	CNN	99.936 ± 0.076 ^ab^	99.031 ± 0.565 ^b^	99.457 ± 0.162 ^a^
deep feature fusion	SVM	100.000 ± 0.000 ^a^	99.922 ± 0.174 ^a^	99.922 ± 0.106 ^a^
	LR	99.987 ± 0.018 ^a^	99.845 ± 0.162 ^a^	99.922 ± 0.106 ^a^
	CNN	99.981 ± 0.029 ^a^	99.884 ± 0.260 ^a^	99.922 ± 0.106 ^a^

The letters (a, b) in each column indicate the significance of the difference in the accuracy of different models at the confidence level of 5%. Within a column, data followed by different letters are significantly different.
